# The fate of bitumen: an exploratory study of national newspaper coverage of Alberta’s bitumen industry during the COVID-19 pandemic

**DOI:** 10.1057/s41599-023-01515-2

**Published:** 2023-01-23

**Authors:** Sibo Chen

**Affiliations:** School of Professional Communication, Toronto Metropolitan University, Toronto, ON Canada

**Keywords:** Cultural and media studies, Environmental studies

## Abstract

This exploratory study examines how three major Canadian newspapers—the *Globe and Mail*, the *National Post*, and the *Toronto Star*—reported on Alberta’s bitumen industry throughout 2020, when the COVID-19 pandemic introduced significant market volatility. Through a combination of computational text analysis and qualitative interpretation, the study identified four recurring themes in 685 articles published by the target newspapers: (1) contention over bitumen infrastructure, (2) economic challenges for the bitumen industry, (3) political divide over the future of bitumen, (4) the bitumen industry’s environmental impacts. A further qualitative assessment of these themes indicates that despite the pandemic’s exposure of the structural weaknesses underlying Canada’s resource-dependent economy, voices supporting the bitumen industry continue to dominate Canadian mainstream newspapers.

## Introduction

The ongoing COVID-19 pandemic has created both opportunities and challenges for climate change mitigation. On the one hand, reduced economic and social activities due to public health measures have led to substantial reductions in both air pollutants and greenhouse gas (GHG) emissions, demonstrating that decarbonization at the global scale is achievable through collective efforts. If an economic recovery that prioritizes renewable energy infrastructure is implemented, the pandemic could serve as a potential tipping point for the planned decline of fossil fuels (Rosenbloom and Markard 2020). On the other hand, the economic downturn along with the pandemic has prompted many national governments to stimulate economic recovery by subsidizing the oil, gas, and coal industries. This could result in “carbon lock-in,” thereby making the transition to a low-carbon future more difficult and costly (Hoang et al. 2021).

Given the contradictory trends surrounding decarbonization, the proportion of COVID-19 recovery expenditures that should be allocated to the fossil fuel sector has become a highly contentious issue in numerous resource-dependent countries. Recent economic policymaking in Canada, for instance, suggests ambivalence over the future of the domestic oil and gas sector, which is the country’s largest greenhouse gas (GHG) emitting sector and a major economic contributor (Statista 2022). While the Canadian federal government has pledged to achieve net-zero emissions by 2050, which would necessitate a significant reduction in fossil fuel production and consumption, it continues to provide subsidies for the oil and gas sector. As a result of COVID-19 stimulus spending, fossil fuel subsidies in Canada increased in 2020 compared to prior years (Corkal and Gass 2020).

The Canadian federal government’s mixed signals regarding the future of the domestic oil and gas sector point to a dilemma that confounds many countries as they struggle to simultaneously revive their economies in the wake of the COVID-19 pandemic and transition to a low-carbon future. Meanwhile, accompanying this dilemma in the public sphere is an alarming upsurge of climate delay discourses, which, by framing climate change as a secondary concern in comparison to the urgent need for economic recovery, poses a significant threat to climate change mitigation (Ecker et al. 2020).

In this context, it is more crucial than ever for environmental communication research to examine the influence of climate delay discourses on public perceptions of climate emergency. This article focuses on the ongoing public debates regarding the future of Canada’s oil-dependent economy in relation to the systemic crises caused by the COVID-19 pandemic. Through an explorative study of major Canadian national newspapers’ coverage of Alberta’s bitumen sector in 2020, it discusses the relationship between climate change and storytelling in an era of political polarization (Arnold 2018).

## Literature review

In recent decades, the Canadian media’s coverage of climate change and the domestic oil and gas sector has been intertwined. Between 1997 and 2010, climate change news in Canada coincided with major national and international turning points in scientific knowledge production and policymaking by exhibiting a series of peaks and troughs (Stoddart et al. 2021). During peak periods (e.g., the year preceding the Copenhagen Climate Summit), the coverage of climate change in major Canadian national newspapers such as the *Globe and Mail* and the *National Post* was dominated by thematic frames such as science and technology, policymaking, and economic and energy interests.

Although these thematic frames have remained prominent in Canadian environmental discourses in recent years, they have been profoundly influenced by growing political contention. Notably, how decarbonization will affect the future of the domestic oil and gas sector has become an increasingly divisive issue in the Canadian public sphere, with the media playing a crucial role in mediating competing claims and narratives. This is evidenced by recent public controversies concerning mega Canadian fossil fuel projects (e.g., the Northern Gateway Pipeline project and the Pacific NorthWest Liquefied Natural Gas project), during which different news outlets disseminated relevant coverage that reflected competing stakeholder concerns (Chen 2020; Dusyk et al. 2018).

Canadian environmental news is also influenced by a variety of local factors, which occasionally overshadow public concern about climate change. During public debates over the Northern Gateway Pipeline project, for instance, Canadian newspapers reported disputes regarding the project’s environmental risks predominantly in terms of potential local impacts (e.g., pipeline or tanker rupture), whereas the impacts of expanding Alberta bitumen production on climate change were rarely mentioned (Dusyk et al. 2018). The Canadian media’s coverage of climate change’s health consequences demonstrates a similar failure to link the local and global impacts of climate change. According to King et al. (2019), this issue only received sustained coverage in Canada’s Northern Territories (Yukon, Northwest Territories, and Nunavut), which may lead to the misconception that climate change’s effects on health will be limited to Arctic regions and may dampen concern. The coverage of climate change by the anglophone and francophone media in Canada differs as well. As Young and Dugan (2012) note, English outlets present a diverse range of coverage that is highly compartmentalized, while French newspapers present a narrower range of coverage but with thematically richer articles that better connect climate change issues to the spheres of culture, politics, and economy.

Despite such regional factors, the mounting urgency of fossil fuel phase-out is currently the most divisive issue among policymakers, political commentators, and ordinary citizens (Carroll 2021). The was manifested in how these stakeholder groups reacted to the 2021 United Nations Climate Change Conference (COP26). To begin with, although the Canadian federal government joined the COP26 pledge of halting investment in coal power generation and phasing out coal entirely by 2040, its stance on the future of Alberta bitumen, the country’s largest crude oil sector with significant environmental impacts, suggested considerable reluctance (Chun 2021). Four months after COP26, it released a new federal emissions reduction plan, calling for a 42% reduction in oil and gas industry emissions to reach Canada’s 2030 emissions reduction goal (Platt 2022). This ambitious goal, however, did not impose a strict limit on the expansion of the bitumen industry in the near future.

To Canadian environmental organizations, COP26 presented a crucial opportunity for urgent and significant action on immediate decarbonization. Accordingly, they responded negatively to the federal emissions reduction plan by noting that it would inevitably fail because of its lax approach to limiting the bitumen industry. In the words of Environmental Defense programs director Keith Brooks, the plan “indulges in magical thinking in proposing that oil production can increase by almost a million barrels per day while emissions come down” (cited in Otis 2022, para. 2).

In contrast to Canadian environmental organizations’ push for rapid and comprehensive decarbonization, the provincial government of Alberta claimed that the federal emissions reduction plan, which expressed the federal government’s reluctance to defend the bitumen industry, disproportionately penalized Albertans (Varcoe 2022 April 4). This was consistent with Alberta’s prior stance on decarbonization. As Canada’s fossil fuel heartland, the province has long been vocal in its defense of the bitumen industry from what it calls “environmental radicals.”

For example, on October 21, 2021—less than two weeks before COP26—the Alberta government released the final report of its two-year inquiry into “foreign funded anti-Alberta energy campaigns”. Although the report found no wrongdoing of any groups or individuals (Cryderman 2021), Alberta Energy Minister Sonya Savage still praised it as an important piece of history, stating that “Albertans should be outraged at the foreign-funded campaigns that targeted our oil and gas sector in an attempt to block development” (“Foreign funding hurt Alberta’s energy development” 2021, para. 2).

Conservative political commentators are Alberta’s most visible media allies. Echoing Savage’s statement, Rex Murphy (2021), a well-known conservative columnist at the *National Post*, denounced Justin Trudeau’s refusal to publicly defend fossil fuels at COP26, claiming that “it is so bad to see Alberta used as a trading card for cheap cheers in service to a specious cause” (para. 13).

The hostility toward decarbonization exemplified by public figures such as Sonya Savage and Rex Murphy is indicative of the profound politicization of climate change in Canadian public discourse. At first glance, the federal government’s position on emissions reduction appears to contradict Alberta’s. Yet, both sides are in fact variants of the “climate delay rhetoric”, which downplays or discounts the need to take immediate and bold action (Lamb et al. 2020). According to Tindall et al. (2021), at the heart of the climate delay rhetoric is “climate change denialism 2.0,” which seeks to obstruct the progress of phasing out fossil fuels in covert and less confrontational ways. Put differently, although transnational oil and gas conglomerates rarely publicly deny the gravity of the climate crisis these days, they consistently advocate for non-transformative solutions and cast doubt on the possibility of restructuring the global economy around renewable energy sources within a decade or two.

Climate change denialism 2.0 has already infiltrated current policy debates about achieving net-zero emissions. Buck (2021) argues that there exist two competing approaches to addressing current greenhouse gas emissions: a “cleaner fossil world” versus a “near zero world.” The former approach will allow for the continuous production of fossil fuels with lower carbon intensity while maintaining net zero emissions through carbon storage and other means of sucking up massive amounts of carbon. In contrast, the latter approach will eliminate the use of fossil fuels in most human activities, necessitating only minor infrastructure construction to generate negative emissions. Climate change denial 2.0, according to Buck’s critical assessment of current policy discussions about “net-zero emissions by 2050,” plays a critical role in legitimizing the “cleaner fossil world” vision, thereby maintaining the current carbon-intensive economic growth model.

The ongoing public debates over the future of Canada’s oil-dependent economy in relation to the prolonged economic downturn brought by the COVID-19 pandemic provide a unique opportunity to analyze whether climate change denial 2.0 has turned the pandemic into an opportunity to justify the necessity of continuing investing in Alberta bitumen. According to Stoddart et al. (2021), COVID-19 has influenced Canadian media coverage of climate change in two ways: it was associated with a period of decreased media attention, but it also created new opportunities for news framing that connects the environmental and economic dimensions of sustainability. Echoing Stoddart and his colleagues’ call for additional research on the disruptive impact of COVID-19 on climate change communication, the current study seeks to determine how the pandemic influenced the coverage of Alberta’s bitumen sector in Canadian national newspapers throughout 2020.

## Data and methods

This study examines bitumen-related news articles published by the *Globe and Mail*, the *Toronto Star*, and the *National Post*, the top three Canadian newspapers by circulation (Agility 2021). It uses a mixed-methods approach that Jacobs and Tschötschel (2019) describe as “topic modeling meets discourse analysis”. The approach begins by reducing the complexity of a corpus by identifying word clusters that reappear across multiple texts (quantitative topic modeling). These word clusters are then interpreted qualitatively using the “keyword-in-context” (KWIC) method (explained later in this section). The empirical analysis is guided by two research questions:

RQ1: How did major national newspapers in Canada report on Alberta bitumen throughout 2020?

RQ2: What overarching narratives concerning the future of Alberta bitumen emerged from these newspapers’ reporting?

I collected relevant articles published by these newspapers from Factiva, using the search string “oil sands OR oilsands OR bitumen OR tar sands OR tarsands” and the publication period “Jan/01/2020 to Dec/31/2020”. With the duplicates setting set to “similar,” the initial search yielded 732 articles.

I then refined the initial dataset by manually checking each article. This procedure sought to remove articles that only made passing references to bitumen. For instance, it was discovered that the initial data collection captured several daily COVID-19 updates published by the *Toronto Star* since they reported COVID-19 cases at bitumen facilities, but their main content was unrelated to the bitumen industry. After removing such articles, the finalized dataset (*N* = 685) consists of 284 articles published by the *Globe and Mail*, 153 published by the *Toronto Star*, and 248 published by the *National Post*.

The data analysis process involved three steps: (1) text preprocessing, (2) Latent Dirichlet Allocation (LDA) topic modeling, and (3) qualitative deep reading of concordance lines of selected keywords. Recent studies such as Hubner (2021) and Nicholls and Culpepper (2021) recommend retaining only the headline (HD) and lead paragraph (LP) of each article when constructing a news corpus for topic modeling. This is based on the premise that it captures the essence of each article in as few words as possible, which enhances the accuracy of LDA. In accordance with the recommendation, I began the text preprocessing step by abridging the collected articles. As the default format of news articles downloaded from Factiva includes a HD section and a LP section, I wrote a Python script to extract texts from then, thereby transforming the original dataset into an abridged corpus. I then completed the text preprocessing step by tokenizing the corpus (i.e., removing stop words, punctuation, and numbers), which is a prerequisite before performing topic detection using techniques like LDA. The current study used the Natural Language Toolkit (NLTK) Python library’s default stop words list.

I conducted the LDA topic modeling step using the open-source python package “Orange Data Mining” (Demšar et al. 2013). LDA is predicated on the following assumptions (Blei et al. 2003): (1) there are a set of topics in a textual document; (2) each topic consists of a collection of words; and (3) topics can be detected quantitatively by analyzing the co-occurrences of words. As LDA is a probabilistic model revealing the probability of each word cluster in each document, its results require further theoretically informed human interpretation.

Additionally, LDA is an unsupervised machine learning method that requires only a predefined k-value (i.e., the number of assumed topics) to classify word clusters in a corpus. The corpus is then analyzed following a process similar to inductive reasoning in grounded theory. In this study, I tested k-values ranging from 5 to 20. After inspecting the outputs of each k-value, I chose a 12-topic model since it yielded the highest *C*_*V*_ topic-coherence score (0.38), which, according to Röder et al. (2015), is particularly suitable for evaluating topic quality. Notably, this score was still slightly below the value recommended by topic-modeling guidelines (see Pedro 2022). This issue was most likely caused by the corpus’ predefined focus on Alberta bitumen, which resulted in inherent semantic similarity between the identified topics. To address the issue, I enhanced the interpretability of the 12 topics by using LDAvis (Sievert and Shirley 2014), a built-in function of Orange Data Mining that adjusts a given topic’s keyword list based on each word’s uniqueness to the topic.

In order to validate and contextualize the topic modeling results, I conducted a KWIC analysis of selected LDAvis-retrieved keywords. For a given keyword, I interpreted its concordance lines in the corpus to better comprehend the discursive patterns, routines, and logics associated with the LDAvis-identified word clusters. The interpretation process was informed by previous literature and the author’s prior research expertise on political contention over bitumen in the Canadian public sphere. For example, the LDA modeling identified the Keystone XL Pipeline Project as a major focus of the corpus. Based on this finding, I searched for headlines and lead paragraphs containing the keyword “keystone” in the corpus to inspect how the target newspapers discussed the pipeline.

## Findings

Table 1 lists the top 30 keywords of the corpus by their term frequency–inverse document frequency (TF–IDF) scores. In line with the search criteria used during data collection, the words ranking at the top of the list are “oil,” “Alberta,” “energy,” and “oil sands,” confirming that after text preprocessing, the corpus still reflects the primary focus of the original dataset. Meanwhile, words such as “pipeline,” “project,” “Teck,” “frontier,” and “mine” (the latter three refer to the Frontier Oil Sands Mine proposed by Teck Resources) suggest that discussions about bitumen in the corpus were likely to center on energy infrastructure. Another word cluster in the list consists of “climate,” “federal,” “Trudeau,” “Ottawa,” and “Kenney” (referring to Alberta Premier Jason Kenney), which, after further inspecting their concordance lines in the corpus, indicates Alberta’s political divide with the federal government over how to regulate the bitumen industry’s carbon emissions. Surprisingly, although “COVID” was included in the search string during data collection, it ranks only 28th in the list.

**Table 1:** Top 30 keywords of the abridged corpus.

Word	TF-IDF
Oil	0.0411
Alberta	0.0321
Energy	0.0301
Canada	0.0285
Oilsands	0.0271
Pipeline	0.0207
Canadian	0.0198
Gas	0.0197
Government	0.0191
Project	0.0189
Teck	0.0186
Climate	0.0173
Per	0.0162
Federal	0.0161
New	0.0151
Industry	0.0150
Said	0.0148
Frontier	0.0144
Would	0.0138
Trudeau	0.0134
Prices	0.0132
Ottawa	0.0132
Covid	0.0130
Cent	0.0130
Emissions	0.0129
Mine	0.0128
Pandemic	0.0128
Says	0.0127
Year	0.0127
Kenney	0.0127

Table 2 presents the twelve topics identified by the LDA topic modeling step. After examining the topic-specific keywords identified by LDAvis and their concordance lines, I assigned a label to each topic. Among these topics, Topic 3 is the most ambiguous for interpretation due to its keywords referring to both the bitumen industry’s environmental impacts (i.e., “methane” and “emissions”) and declining bitumen production due to the COVID-19 pandemic (i.e., “operations,” “oil,” and “downturn”). By contrast, the other topics have identifiable foci and can be classified into four interrelated themes: (1) contention over bitumen infrastructure, (2) economic challenges for the bitumen industry, (3) political divide over the future of bitumen, (4) the bitumen industry’s environmental impacts. Below, I summarize the gist of each theme based on a qualitative interpretation of the concordance lines of distinct keywords (e.g., “downturn,” “emissions,” “trans mountain,” etc.) in the corpus.

**Table 2:** Top-weighted words of topic modeling results.

Theme	Topic Label	Topic-specific Keywords (enhanced by LDAvis)
*Contention over bitumen infrastructure*	2. Trans Mountain Pipeline Expansion	pipeline, oil, one, mountain, trans, Canada, Alberta, economic, energy, pandemic, price, federal, Ottawa, oil sands, victims, government, would, TMX, COVID, shares
	11. Frontier Oil Sands Mine	project, Alberta, Teck, frontier, oil sands, oil, federal, government, decision, mine, court, proposed, pipeline, approval, gas, Ottawa, Itd, resources, Canada, Kenney
	12. Keystone XL	Keystone, XL, U, Toole, next, pipeline, Biden, leadership, Erin, campaign, Joe, permit, approvals, presumptive, space, energy, year, stories, elected, candidate
*Economic challenges for the bitumen industry*	5. Oil supply and demand	per, oil, cent, energy, oil sands, Canada, gas, crude, new, Inc, Canadian, Alberta, environmental, would, demand, Suncor, emissions, Calgary, producer, natural
	6. Mergers and acquisitions within the bitumen industry	Waterous, Osum, bid, fund, Canada, gas, oil, million, request, halt, stake, hostile, Buffalo, Wef, marketing, economy, Trump, Alberta, including, pursue
	7. Shifts in bitumen investment	oil, Canada, climate, industry, change, Jarislowsky, investment, plan, Carney, foreign, pension, companies, new, back, energy, war, divestment, Alberta, England, precision
	10. Market volatility	energy, oil, sector, pipeline, futures, rebound, price, demand, technology, storage, Canadian, provinces, earths, join, stocks, giant, small, supply, reopen, pandemic
*Political divide over the future of bitumen*	1. Indigenous issues in energy politics	oil, oil sands, world, Indigenous, Canadian, energy, back, Canada, Meis, next, cent, per, McKay, country, group, first, street, production, Alberta, traders
	8. Political divide over the bitumen industry	energy, Alberta, Canada, said, government, Trudeau, oil, oil sands, country, province, Husky, Cenovus, coal, infrastructure, federal, massive, deal, protests, Justin, Wet
*The bitumen industry’s environmental impacts*	4. The environmental impacts of bitumen	Moe, ice, Jean, Saskatchewan, Moore, party, Canada, last, Athabasca, report, ponds, tailings, evidence, groundwater, leaking, Hangingstone, says, country, bill, blockades
	9. Mitigating GHG emissions	climate, oil, nuclear, Canada, gas, spending, years, emissions, change, targets, economy, net, year, Cenovus, per, energy, prices, billion, cent, industry
*Ambiguous for interpretation*	3. Environmental issues + economic downturn	oil, million, Alberta, Bezos, emissions, COVID, Kenney, operations, says, gas, methane, premier, per, complex, public, loss, inter, comments, points, cent

### Contention over bitumen infrastructure

Given the prevalence of infrastructure-related keywords in Tables 1 and 2, the qualitative analysis began by examining how the unbridged dataset addressed bitumen projects such as the Trans Mountain Pipeline Expansion and the Frontier Oil Sands Mine. Although the corpus provided regular updates on contention over bitumen infrastructure throughout 2020, the issue received the most coverage in January and February, prior to the pandemic-induced economic downturn. In such coverage, featured prominently were the voices of the “petro-bloc”—Alberta politicians, bitumen advocates, and corporate representatives, who collectively depicted the bitumen industry as negatively impacted by market volatility, regulatory hurdles, and environmental opposition.

For example, when Teck Resources withdrew its Frontier Oil Sands Mine proposal at the end of February, bitumen advocates framed the decision as an indicator of “the beginning of Canada’s irreversible economic decline caused by the anti-enterprise policies of Prime Minister Justin Trudeau’s regime” (Francis 2020, para. 3). Echoing this opinion, similar sentiments were expressed regarding other stalled bitumen projects in the dataset. Collectively, such critiques on the Trudeau government’s “anti-enterprise policies” advocated for rescuing the bitumen industry from a “regulatory and legal morass” (Snyder 2020, para. 5).

### Economic challenges for the bitumen industry

Along with the unprecedented energy market slump imposed by the pandemic, the urgency and necessity of salvaging the bitumen industry became a major focus of the dataset. As a result, bitumen proponents began to talk less about deregulation and instead discussed government intervention in terms of subsidies and preferential policies. The indispensable role of the bitumen industry in providing employment opportunities emerged as a recurring justification in articles demanding public funding for it, which, however, is controlled by private and transnational capital. Consider, for example, the following narrative from Grace Richards, a heavy equipment operator quoted in a report titled “Faces of the Economic Fallout” (2020 March 21):

I’m used to making good money, but now I’ve been trying to call about my employment insurance. I’ve been trying to call my bank about my vehicle payment. I’ve been trying to call my insurance company, and I can’t get through to anybody. I’m kind of getting anxiety over this. (para. 4)

Yet, absent from discussions on the plight of ordinary workers like Ms. Richards were alternatives to rejoining the bitumen industry after the economic downturn. Consequently, bitumen corporations were implicitly positioned as the only viable option for addressing Alberta’s post-pandemic economic recovery.

### Political divide over the future of bitumen

As for energy politics, the dataset primarily featured the policy disputes between the federal government—which advocates for a balanced approach to bitumen development—and the petro-bloc—which seeks to delay additional regulatory measures on the bitumen industry. The near future of bitumen was a focal point of contention for both sides. Take Alberta Premier Jason Kenney’s stance on climate change as an example. He acknowledged for the first time in February 2020 that, in order to combat climate change, the bitumen industry would inevitably decline (Boyd and Leavitt 2020). Nonetheless, he also emphasized that “tens of millions of barrels (of oil) will be consumed before demand decreases, and he wants Alberta to supply them” (para. 5).

Other petro-bloc members echoed Kenney’s climate delay rhetoric by criticizing the federal government’s approach to carbon pricing. As corporate economist Mintz (2020) argued in a representative piece of such criticisms, the federal government’s carbon pricing plan “generates lots of revenue that will be rebated to households, not businesses” (para. 13), thereby damaging the competitiveness of businesses that are either energy-consuming or getting no rebate.

Notably, however, the federal government’s carbon pricing plan—which the petro-bloc deemed as “industry unfriendly”—did not propose a timeline of phasing-out bitumen production. After reviewing the plan’s related coverage in the dataset, it became evident that embedded in the federal government’s various statements on energy transition was a strategic ambiguity regarding whether Canada would head towards a “cleaner fossil world” or a “near zero world” (Buck 2021).

### The bitumen industry’s environmental impacts

Contrary to the federal government and the petro-bloc, environmentalists and their allies in the dataset diagnosed the pandemic-induced economic crisis as a revealing moment of the underlying flaws of Canada’s resource-dependent economy. In accordance with this diagnosis, they called for expanding Canada’s green economy, thereby making its economic recovery more resilient. The *Toronto Star*, due to its center-left political leaning, published numerous pieces in support of the resilient recovery idea. In contrast, the *Globe and Mail* and the *National Post* marginalized environmental voices, notably in reports appearing at their business sections.

In the dataset, the attacks on Elizabeth May—the then leader of the federal Green Party—by the petro-bloc presented a high-profile incident exemplifying how calls for decarbonization were discredited. May stated during a news conference in May 2020 that “oil is dead.” Echoing the climate delay rhetoric discussed in Ecker et al. (2020), the petro-bloc’s responses to May’s statement framed fossil fuel phase-out during an unprecedented economic collapse as “mission impossible.” In an editorial titled “Canadian oil’s exaggerated death” (2020), for instance, the *National Post* problematized May’s stance by employing two prevalent pro-bitumen discursive strategies (Carroll 2021): (1) defining fossil fuel consumption as a life necessity and (2) emphasizing the importance of bitumen development for Canada’s energy self-sufficiency.

### Concluding remarks

When confronted with an unprecedented crisis like the COVID-19 pandemic, citizens typically resort to the media for information and expert opinions. Accordingly, the public perceptions of pandemic-related issues are likely to be influenced by relevant news coverage. This exploratory study seeks to comprehend how COVID-19 has reshaped energy communication and media framing in the Canadian context. For this purpose, it examines how mainstream Canadian newspapers—as represented by the *Globe and Mail*, the *National Post*, and the *Toronto Star*—discussed the bitumen industry’s bleak outlook throughout 2020.

In response to RQ1 and RQ2, the mixed-methods analysis revealed that the target newspapers portrayed COVID-19 as a primary factor disrupting global energy market, thereby placing bitumen corporations in an existential crisis. Largely marginalized in this crisis narrative were pro-climate perspectives that view COVID-19 and climate change as parallel crises requiring coordinated responses. This was particularly evident in the business reports published by the *Globe and Mail* and the *National Post*, in which discussions on the economic challenges for the bitumen industry underscored a collective plea for “returning to normal”. In light of the energy sector’s importance to Canadian economic policymaking prior to the pandemic, this plea contradicted the mounting public calls for diverting pandemic subsidies away from the bitumen industry. While such calls for green alternatives received some coverage in a number of articles, the pervasive pro-bitumen narratives of the petro-bloc have nullified their discursive power.

Thus, despite the vulnerability of Canada’s resource-dependent economy exposed by the pandemic-induced energy market volatility, energy discourses in mainstream Canadian media continue to view decarbonization as a secondary concern compared to restoring pre-pandemic economic normalcy. This is consistent with previous research on climate change and media issue attention cycles (e.g., Chen 2020; Dusyk et al. 2018; Stoddart et al. 2021), which has found that prominent domestic and international political events frequently divert public attention away from climate change mitigation, notwithstanding the issue’s growing urgency. In the current case, the pro-bitumen narratives provided economic and political justifications for climate change denialism 2.0 (Buck 2021, Tindall et al. 2021). Central to these justifications was the petro-bloc’s efforts to define the bitumen industry’s future growth as integral to Canada’s public interest, thereby denying the urgency and feasibility of a quick fossil fuel phase-out. Given the detrimental impact of climate delay rhetoric on public support for decarbonization (Ecker et al. 2020; Lamb et al. 2020), it is concerning that economic arguments against climate action remain prevalent in Canadian media coverage of the bitumen industry.

This study is constrained by the fact that its evaluation of the collected news articles was based on an abridged corpus. Consequently, its findings could benefit from more nuanced, but resource-intensive, in-depth readings of a smaller set of news texts. Despite being exploratory, however, this study makes three contributions to the field of environmental communication. Theoretically, by explicating media coverage of the post-pandemic economic recovery in the Canadian context, it adds to ongoing scholarly discussions on how COVID-19 has altered the post-Paris trajectory of climate policy. Methodologically, the study demonstrates the benefits of using topic modeling, a technique that has gained popularity among discourse scholars (Jacobs and Tschötschel 2019), to systematically identify word clusters for qualitative KWIC analysis. Practically, the study urges increased public scrutiny of the economic and political legitimacy of pandemic subsidies.

In conclusion, this article has provided new insights into the ongoing discursive struggles over energy transition. Given the continuing dominance of the petro-bloc’s voices in major Canadian news outlets such as the *Globe and Mail* and the *National Post*, there is an urgent need for environmental groups and their allies to explore alternative communication channels to engage the public with inspiring stories about the promises of the green economy. Accordingly, future research could build on the current study by evaluating the potential of new media (e.g., TikTok, Instagram, and podcasts) in facilitating public conversations about decarbonization.

## Data Availability

The raw data supporting the conclusions of this study are available on request to the corresponding author.
